# LKB1 Mediates the Development of Conventional and Innate T Cells via AMP-Dependent Kinase Autonomous Pathways

**DOI:** 10.1371/journal.pone.0060217

**Published:** 2013-03-22

**Authors:** Marouan Zarrouk, Julia Rolf, Doreen Ann Cantrell

**Affiliations:** Division of Cell Signalling and Immunology, College of Life Sciences, University of Dundee, Dundee, Scotland, United Kingdom; Oklahoma Medical Research Foundation, United States of America

## Abstract

The present study has examined the role of the serine/threonine kinase LKB1 in the survival and differentiation of CD4/8 double positive thymocytes. LKB1-null DPs can respond to signals from the mature α/β T-cell-antigen receptor and initiate positive selection. However, in the absence of LKB1, thymocytes fail to mature to conventional single positive cells causing severe lymphopenia in the peripheral lymphoid tissues. LKB1 thus appears to be dispensable for positive selection but important for the maturation of positively selected thymocytes. LKB1 also strikingly prevented the development of invariant Vα14 NKT cells and innate TCR αβ gut lymphocytes. Previous studies with gain of function mutants have suggested that the role of LKB1 in T cell development is mediated by its substrate the AMP-activated protein kinase (AMPK). The present study now analyses the impact of AMPK deletion in DP thymocytes and shows that the role of LKB1 during the development of both conventional and innate T cells is mediated by AMPK-independent pathways.

## Introduction

The adaptive immune response is mediated by T cells that express T cell antigen receptor complexes comprising of highly variable TCRα and β subunits [1]. These T cells can be subdivided into cells that express CD8, the receptor for major histocompatibility antigen complex I (MHC class I), and cells that express CD4, the receptor for MHC class II molecules. CD4 positive T cells can be further subdivided into conventional CD4 T cells, regulatory T cells (Tregs) and Natural Killer T (NKT) cells [2]. Conventional CD4 and CD8 T cells express α/β TCR complexes that recognize peptide/MHC complexes whereas NKT cells express an invariant Vα14 T cell receptor that recognize glycolipid/CD1d antigen complexes (iNKTs) and play a role in immune surveillance and immune homeostasis [3]. CD8 T cells can also be subdivided into conventional CD8 cells that express a CD8 αβ heterodimer and CD8 T cell populations that express a CD8αα homodimer [4]. TCRαβ^+^ CD8αβ^+^ conventional T cells recirculate between the blood, secondary lymphoid tissue and the lymphatics and respond to immune activation and differentiate to produce cytolytic effector cells. TCRαβ^+^ CD8αα^+^ T cells are typically found in the epithelial layer in the gut and play a role in regulating inflammatory immune responses in the gut [5].

The balanced production of different T cell subpopulations, each with unique functions, during thymus development is essential to ensure the function and the homeostasis of the peripheral immune system. Hence, understanding the nature of the signals required for the development of different T cell subpopulations is important. All T cells that express αβ TCR complexes develop in the thymus from progenitors that lack expression of CD4 and CD8, hence termed double negative (DN) thymocytes. At the DN stage of thymocyte development T cell progenitors undergo genetic rearrangement of the TCRβ locus, which leads to the expression of a pre-TCR complex. This immature TCR complex drives DNs to proliferate and differentiate into CD4/8 double positive (DP) thymocytes. DP thymocytes that have successfully re-arranged their TCRα chain will undergo a selection process and differentiate to conventional TCR αβ CD4^+^ or CD8^+^ T cells, NKT cells or TCRαβ^+^ CD8αα^+^ gut lymphocytes.

In this context, there is currently considerable interest in understanding the signalling pathways that control metabolic checkpoints in T lymphocytes. It is thus relevant that recent studies have shown that the serine/threonine kinase LKB1 (Liver kinase B1 also known as serine/threonine kinase 11 - STK11) is important in controlling metabolic homeostasis in early T cell progenitors in the thymus [6], [7]. There is also evidence that LKB1 is important in CD4/CD8 DPs. LKB1 null DPs thus appear to be unable to develop into conventional TCRα/β CD4^+^ and CD8^+^ T cells [8], [9]. However, there are a number of important unanswered questions about LKB1 and its role in thymus development. For example, is LKB1 required for DP thymocyte survival and does this explain why LKB1 null DPs cannot produce mature SP T cells? To date most studies of LKB1 in DP thymocytes have studied the few DPs that survive LKB1 deletion at the thymocyte progenitor stage and have not looked at the immediate impact of LKB1 loss in DPs. One other question is whether LKB1 is important in non-conventional T cells, i.e. TCRαβ^+^ CD8αα^+^ IELs or TCRαβ^+^ CD4^+^ iNKTs? In this respect it is evident that LKB1 is not essential for all T cells. For example, LKB1 has an obligatory role to control survival of T cell progenitors [6], [7] but is not essential for the metabolic control of quiescent naive T cells in the periphery [6]. One other fundamental question is how does LKB1 control T cell development? One proposal is that LKB1 controls thymocyte development via regulation of the adenosine monophosphate (AMP)-activated protein kinase α1 (AMPKα1) [7]. This kinase is phosphorylated and activated by LKB1 in response to cellular energy stresses that cause increases in cellular AMP:ATP ratios [10]. It is a candidate to mediate the role of LKB1 in thymocyte development because in many cell lineages AMPKα1 acts to restore cellular energy balance by terminating ATP consuming processes and stimulating ATP generating pathways [10]. However, the evidence supporting a role for AMPKα1 in thymocyte development stems solely from experiments where overexpression of a constitutively active AMPKα1 construct could promote survival of LKB1 null DP thymocytes [7]. This gain of function strategy does not inform whether AMPKα1 is essential for thymus development. It is thus relevant that mice homozygous for deleted AMPKα1 alleles appear to undergo normal thymocyte development [8], [11]. The caveat of these studies is that AMPKα1 null mice on a mixed genetic background are not born at normal Mendelian frequency and indeed global deletion of AMPK results in embryonic lethality on a C57Bl/6 background [11].

The studies to date about the role of AMPKα1 in T cells have thus been on the few mice that can compensate AMPKα1 loss in early embryo development. Accordingly, to directly compare the impact of AMPKα1 deletion and LKB1 deletion on thymus development there is a requirement to compare the consequences of selective deletion of either of these kinases at a defined stage of thymus development. We have therefore used a CD4Cre transgene to delete LKB1 or AMPKαl floxed alleles at the DP stage of thymocyte development. We found that LKB1 does not regulate survival of DP thymocytes although these cells fail to differentiate to conventional TCRα/β SP cell populations and are also defective in the development of iNKT cells and TCRα/β CD8αα IELs. In contrast, AMPKα1 null DPs produce normal numbers of both conventional and innate TCR αβ peripheral T cells. LKB1 is thus essential for the development of both conventional and innate TCR αβ T cells in the thymus but its mode of action is not through the activation of AMPK.

## Results

### DPs survive without LKB1

**Figure 1:**
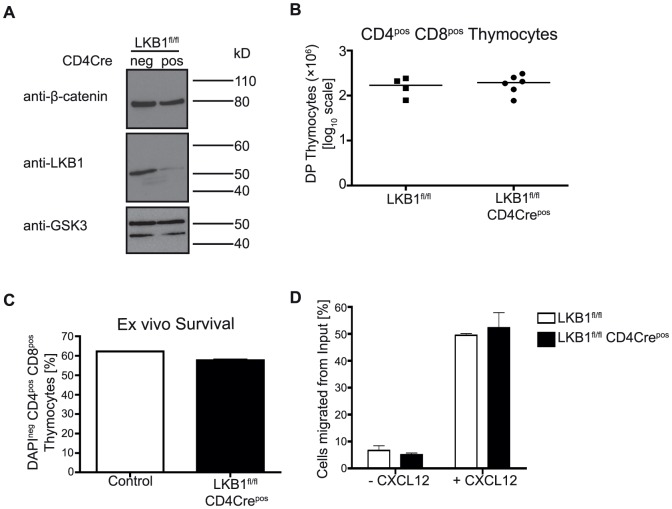
Double positive thymocytes survive without LKB1. (A) CD4^pos^ thymocytes purified from LKB1^fl/fl^ CD4Cre^neg^ or CD4Cre^pos^ thymi using MACS were lysed at 3×10^7^ cells mL^−1^ in lysis buffer. Proteins extracted from cells and denatured were resolved on NuPAGE Bis-Tris 4–12% gels under reducing conditions and subsequent immunoblots were probed for indicated proteins, showing that LKB1 protein was efficiently deleted. GSK3 and β-catenin were used as loading controls for equal loading. Data are representative of two independent experiments. (B) Freshly isolated thymi from LKB1^fl/fl^ CD4Cre^neg^ or LKB1^fl/fl^ CD4Cre^pos^ mice were mashed to single cell suspensions. Cell number of double positive (DP) thymocytes was determined from a given volume using calibrated counting beads and from the frequency of cells co-stained for the MHC-receptors CD4 and CD8 of total number of thymocytes. Data are summary of four to six mice, where each symbol represents one mouse. (C) Single cell suspensions of freshly isolated thymi from LKB1^fl/fl^ CD4Cre^pos^ or littermate controls were seeded at a cell density of 5−10×10^6^ cells mL^−1^ in complete culture medium for 24 h. Frequency of live DP thymocytes was determined by staining for surface co-expression of CD4 and CD8 and exclusion of cells positive for the DNA binding dye DAPI. Statistical analysis using the Mann-Whitney test showed comparable frequencies of DAPI^neg^ DP cells between LKB1^fl/fl^ CD4Cre^pos^ and controls. Data summarise three independent experiments. (D) Thymocytes were isolated and 1×10^6^ cells placed into the fibronectin-coated upper chamber of the transwell plate. Cells were left to migrate into the lower chamber containing medium only or 500 ng mL^−1^ CXCL12 for three hours. Cells from the lower chamber were collected and counted using counting beads using flow cytometry and the frequency of cells migrated was determined against the input that was used as putatively maximal migration capacity. Data summarise three independent experiments showing mean ± SEM.

To explore the role of LKB1 in DP thymocytes, we backcrossed LKB1^fl/fl^ mice to mice that express Cre recombinase under the control of the CD4 promoter. In this model, *cre* recombinase is expressed during the transition of DNs to DPs and this ensures deletion of LKB1 in DP thymocytes **(** **Figure 1A** **)**. We noted that there appeared to be some residual LKB1 protein in DP thymocytes probably reflecting some asynchrony of LKB1 loss as thymocytes make the DN to DP transition. LKB1 controls the survival of DN thymocytes [6]–[8]. It was also suggested that LKB1 null DPs had survival defects [7]. However, our results indicate that the direct deletion of LKB1 in DP thymocytes did not cause cell death of DPs *in vivo*. LKB1^fl/fl^ CD4Cre^pos^ mice thus have normal numbers of DPs and there was no evidence for increased apoptosis of these LKB1 null DPs **(** **Figure 1B** **)**. Normal DP thymocytes undergo apoptosis if removed from the thymus and cultured *in vitro* in the absence of thymic stroma. The deletion of LKB1 did not increase the rate at which DP cells die when removed from the thymus **(** **Figure 1C** **)**. LKB1 is thus not essential for survival of DP thymocytes *in vivo* or *ex vivo*. A further indication of the viability of LKB1 null DP thymocytes comes from analysis of their ability to respond normally to chemotactic stimuli. DP thymocytes express the chemokine receptor CXCR4 and can chemotax on an integrin matrix in response to CXCL12 (SDF-1α) [12]. The data show that LKB1-deficient thymocytes migrated normally on fibronectin-coated transwells **(** **Figure 1D** **)**. These data indicate that the CXCL12/CXCR4 signalling axis and integrin-dependent adhesion do not require LKB1. They also confirm the viability of LKB1 null DP thymocytes.

### Deletion of LKB1 impairs the production of mature α/β T cells

**Figure 2:**
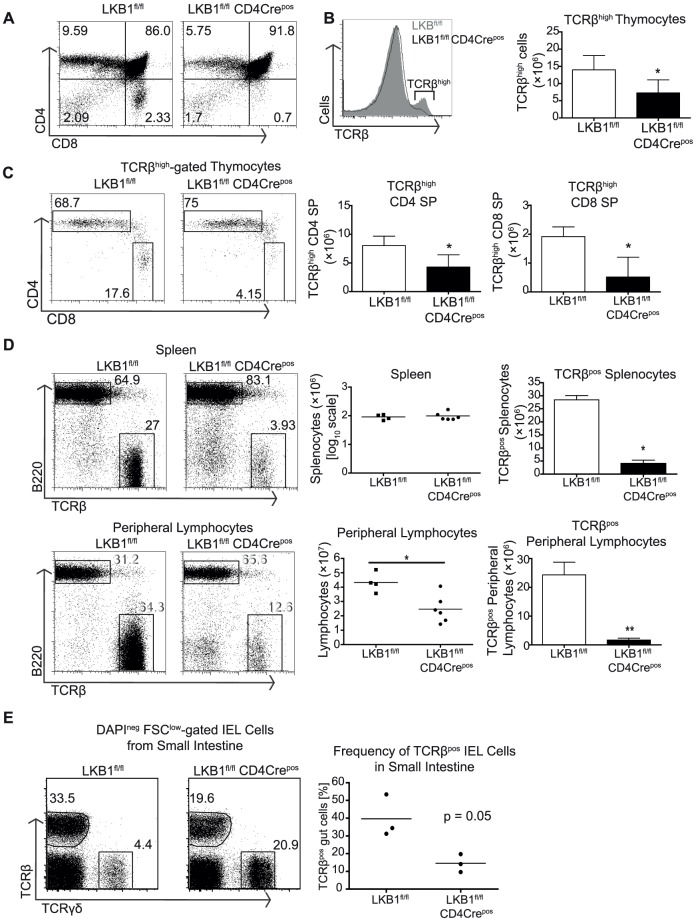
LKB1 required for accumulation of T cells in thymus, lymphoid organs and the small intestine. LKB1^fl/fl^ CD4Cre^neg^ and CD4Cre^pos^ thymi were isolated and analysed for (A) co-expression of CD4 and CD8 and (B) TCRβ expression. (B) Total number of TCRβ^high^ expressing thymocytes was quantified. (C) Ratio and total cell number of CD4^pos^ and CD8^pos^ TCRβ^high^ (SP) cells. (D) Analysis of lymphocyte populations in secondary lymphoid organs (spleen and lymph nodes). The frequency of B cells and T cells was determined by flow cytometric analysis for the expression of B220 and TCRβ, respectively. Quantification of total lymphocytes and TCRβ^pos^ lymphocytes is shown to the right showing that T cells were significantly reduced in secondary lymphoid tissues in LKB1^fl/fl^ CD4Cre^+^ mice. Flow cytometric histograms and plots are representative of four experiments. Dot plots and bar graphs summarise data from at least four independent experiments. (E) Bi-parametric histogram shows frequency of TCRβ^pos^ and TCRγδ^pos^ T cells isolated from the epithelial layer of small intestines from LKB1^fl/fl^ CD4Cre^neg^ and CD4Cre^pos^ mice followed by flow cytometric analysis. Data shown were gated on DAPI^neg^ cells to identify live cells that remained intact following extraction and staining procedures. Dot plot summarises the frequencies of TCRβ^pos^ intraepithelial lymphocytes (IEL) from three mice per genotype. Statistical differences as indicated were determined using the Mann-Whitney test, where *p<0.05 and **p<0.01.

LKB1^fl/fl^ CD4Cre^pos^ mice had normal numbers of DP thymocytes but produced fewer TCRβ^high^ mature CD4 and CD8 SP thymocytes **(** **Figure 2A**–**C** **).** The impact of LKB1 loss on the production of CD8 SP thymocytes appeared more severe than the impact on CD4 T cells **(** **Figure 2A and C** **)**. LKB1^fl/fl^ CD4Cre^pos^ mice also lacked the normal complement of mature αβ CD4 and CD8 SP cells in secondary lymphoid organs such as the spleen and lymph nodes **(** **Figure 2D** **)**. They also did not have a normal frequency of αβ TCR intraepithelial T cells in the small intestine **(** **Figure 2E** **)**.

The transition of DPs to SPs can be staged by expression of the cell surface antigen CD69 and by the levels of TCR αβ complex expression [13]. DP thymocytes thus express low level of TCRβ chains and no CD69. If they undergo a successful rearrangement of their TCR alpha locus and express an αβ TCR complex that recognises self peptide MHC complexes in the surface of thymic epithelial cells they are positively selected and either down-regulate CD4 or CD8 molecules and differentiate to SPs. The first indication of successful TCR engagement in DPs is up-regulation of CD69. Cells undergoing selection then up-regulate expression of αβ TCR complexes [13]. The expression of CD69 is then down-regulated while TCRβ expression remains high on the most mature SPs [13].

**Figure 3:**
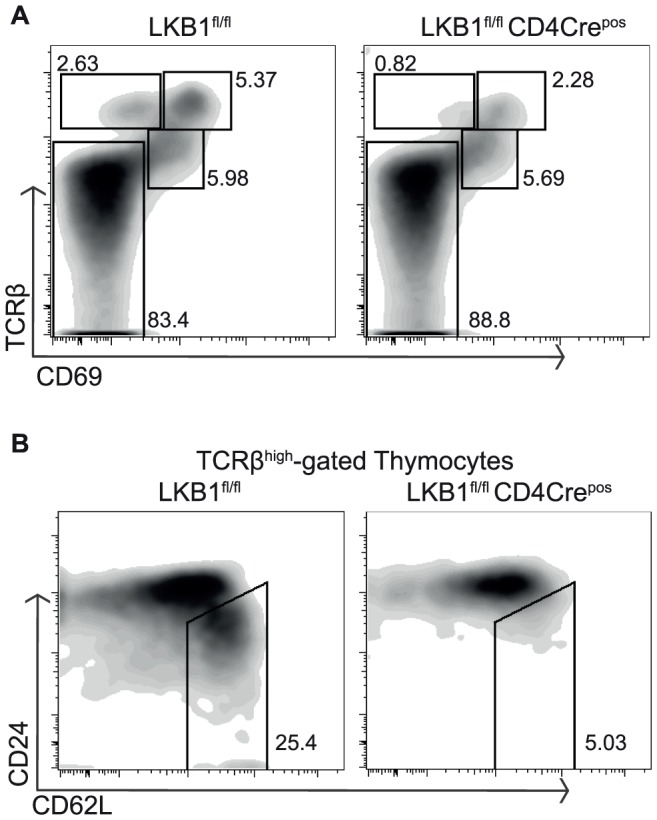
LKB1 is required for the progression of positive selection and maturation of T cells. (A) Thymi from LKB1^fl/fl^ CD4Cre^neg^ and CD4Cre^pos^ mice were analysed for the co-expression of TCRβ and CD69 using flow cytometry as previously described [6], [7], [13]. (B) Flow cytometric analysis of TCRβ^high^ cells stained for the surface expression of CD24 (heat stable antigen) and CD62L (L-selectin). Data shown are representative of at least three mice.

The analysis of CD69 and TCR levels on thymocytes from the LKB1^fl/fl^ CD4Cre^pos^ mice shows that LKB1 null DP thymocytes respond to TCR triggering to up-regulate CD69 expression **(** **Figure 3A** **)**. However, thymocytes co-expressing high levels of both TCRβ and CD69 are reduced approximately by 50% in LKB1^fl/fl^ CD4Cre^pos^ thymi. Mature SP thymocytes down-regulate expression of CD24 but increase expression of the adhesion molecule CD62L (L-selectin). In this context, CD24^low^ CD62L^high^ SP cells were almost undetectable in LKB1^fl/fl^ CD4Cre^pos^ thymi **(** **Figure 3B** **)**. These data show that LKB1 is not required for the TCR mediated signalling events that initiate positive selection but LKB1 null thymocytes cannot complete positive selection to produce mature αβ TCR SP thymocytes. LKB1^fl/fl^ CD4Cre^pos^ thymocytes thus show defective maturation of positively selected SPs rather than a defect in positive selection per se. This explains why LKB1^fl/fl^ CD4Cre^pos^ mice lack mature αβ T cells in peripheral tissues.

### LKB1 is required for NKT cell development

**Figure 4:**
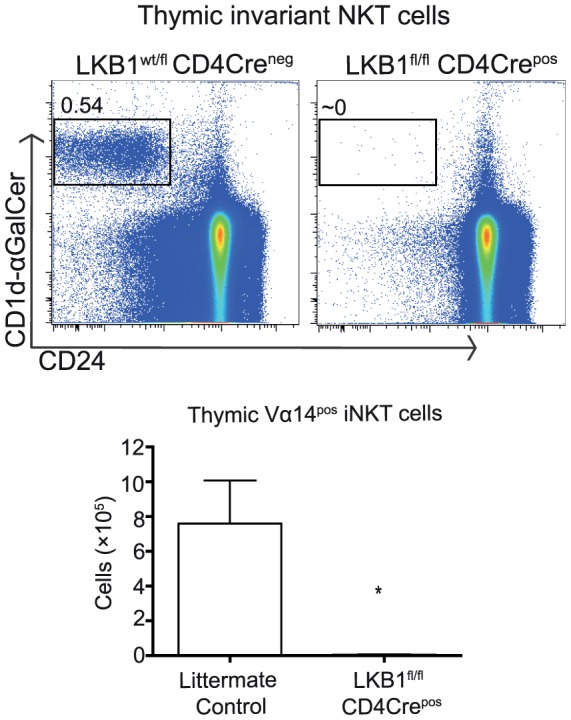
LKB1 is required for the accumulation of thymic iNKT cells. Thymocytes isolated from LKB1^fl/fl^ CD4Cre^pos^ and littermate controls were stained for the presence of invariant NKT cells as described previously [6], [14]. iNKT cells were identified as CD24^low^ CD1d-αGalCer^pos^ thymocyte population. Flow cytometric histograms and frequencies shown are representative of at least three independent experiments. Bar graph summarises total number of CD24^low^ CD1d-αGalCer^pos^ thymocytes from three mice. Statistical difference between groups as shown was determined using Mann-Whitney test where * p<0.05.

DP thymocytes also differentiate to produce CD4^+^ NKT cells that have an invariant Vα14 T cell receptor that recognises glycolipid antigens presented by the MHC-like molecule CD1d. In this context, there is evidence that there are different signalling requirements for the differentiation of iNKT cells and CD4 or CD8 SP mature T cells. For example, DP thymocytes lacking expression of Phospholipid-dependent kinase 1 (PDK1) fail to produce iNKT cells despite normal development of conventional α/β T cells [14]. Also, DP thymocytes that fail to express c-myc fail to develop TCRαβ^+^ CD8αα^+^ IELs or TCRαβ^+^ CD4^+^ iNKTs although they develop conventional TCRαβ^+^ CD4 and CD8 T cells [15], [16]. Do iNKT cells need LKB1 to develop? iNKT cells can be distinguished from conventional αβ T cells as they can bind CD1d molecules loaded with the iNKT cell antigen α-galactosylceramide (αGalCer). **Figure 4** shows that the frequency and total numbers of CD1d-αGalCer^pos^ iNKT cells in control and LKB1^fl/fl^ CD4Cre^pos^ thymi were significantly different. These data show that iNKT cells cannot develop in the absence of LKB1.

### T cell development is independent of AMPK

**Figure 5:**
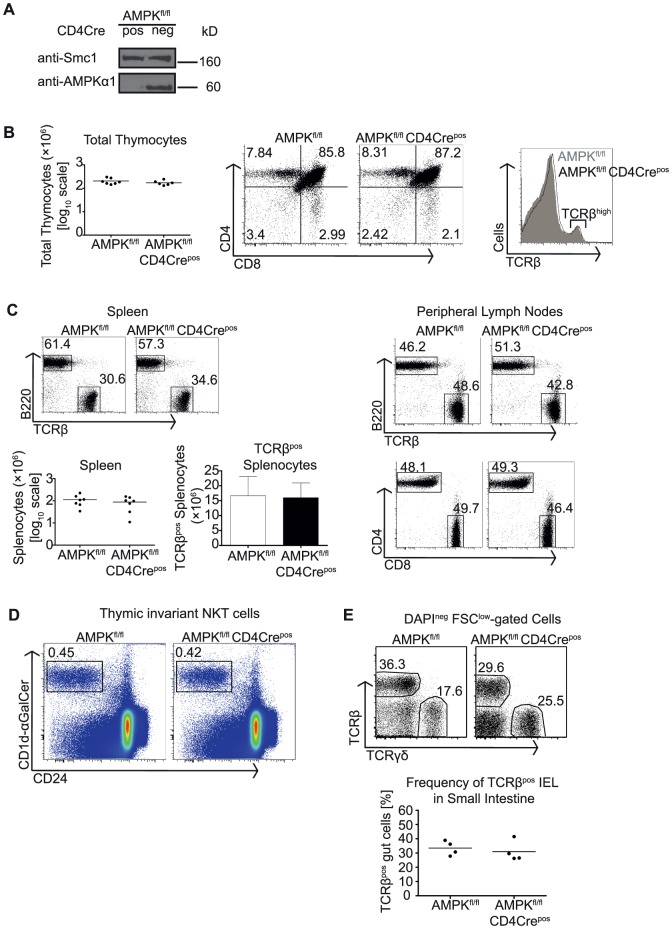
Development and accumulation of T cells does not require AMPKα1. (A) CD4^pos^ thymocytes from AMPKα1^fl/fl^ CD4Cre^neg^ or CD4Cre^pos^ thymi were purified and lysed as described in Figure 1. Immunoblots were probed for AMPKα1 and Smc1 as control for equal loading. Data are representative of at least two independent experiments. (B and C) Thymi and secondary lymphoid organs were isolated from AMPKα1^fl/fl^ CD4Cre^neg^ and CD4Cre^pos^ mice and analysed as described in Figure 1. Quantification of cell numbers in thymi (B) and spleens (C) are also shown. Histograms are representative for at least three mice in panels B and C. Numeric dot plots and bar graphs summarise data from at least five to six mice. Mice were analysed between 60–80 days of age. (D) iNKT cells were identified as CD24^low^ CD1d-αGalCer^pos^ thymocytes isolated from AMPKα1^fl/fl^ CD4Cre^neg^ or CD4Cre^pos^ thymi. Flow cytometric histograms and frequencies shown are representative of at least three independent experiments. (E) TCRβ^pos^ and TCRγδ^pos^ T cells were identified from cell preparations isolated from the epithelial layer of small intestines from AMPKα1^fl/fl^ CD4Cre^neg^ and CD4Cre^pos^ mice followed by flow cytometric analysis as described in Figure 2. Dot plot summarises frequencies of TCRβ^pos^ IEL cells from four different mice. Histograms are representative of four independent analyses.

LKB1 phosphorylates and activates AMPK [6].We therefore interrogated whether the thymic and peripheral T cell phenotype of LKB1^fl/fl^ CD4Cre^pos^ mice was dependent on LKB1-mediated regulation of AMPK. T cells exclusively express the AMPK α1 catalytic subunit and we therefore examined thymus development in AMPKα1^fl/fl^ CD4Cre^pos^ mice. Western blot analysis confirmed that the DP thymocytes that develop in AMPKα1^fl/fl^ CD4Cre^pos^ mice had deleted AMPKα1 **(** **Figure 5A** **)**. However, thymocyte numbers, the production of mature CD4 and CD8 SP T cells in the thymus and the peripheral T cell compartment was normal in AMPKα1^fl/fl^ CD4Cre^pos^ mice **(** **Figure 5B and C** **)**. We also found that the frequencies of iNKT cells in AMPK^fl/fl^ CD4Cre^pos^ mice were comparable to littermate controls **(** **Figure 5D** **)**. The intraepithelial T cell compartment was also normal in AMPK^fl/fl^ CD4Cre^pos^ mice **(** **Figure 5E** **)**.

**Figure 6:**
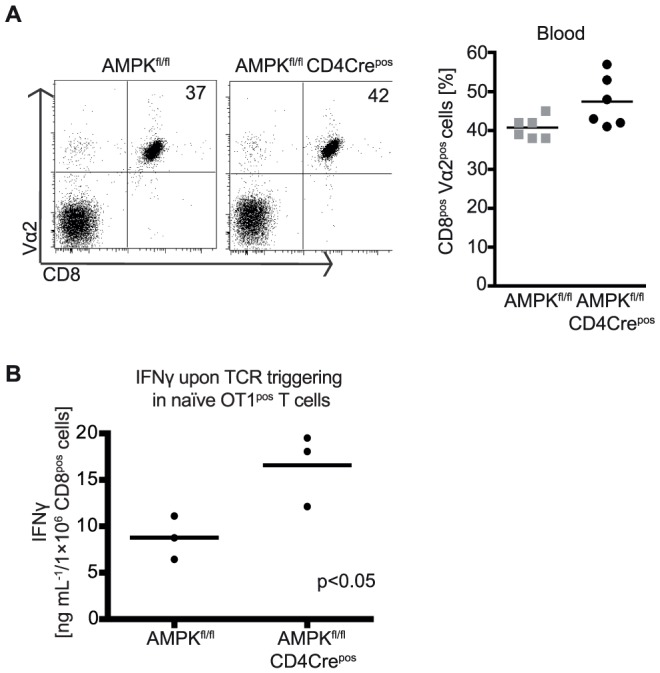
AMPK is not required for T cell development in a TCR-transgenic model. (A) Blood biopsies were immune-phenotyped for the co-expression of the Vα2 TCR chain and CD8 and the frequency of Vα2^pos^ CD8^pos^ cells was determined in OT-1 TCR^pos^ AMPKα1^fl/fl^ CD4Cre^neg^ and CD4Cre^pos^ mice (n = 6). (B) Equal numbers of CD8^pos^ OT-1 TCR^pos^ AMPK^fl/fl^ CD4Cre^neg^ and CD4Cre^pos^ lymphocytes were activated with 0.5 µM SIINFEKL peptide for 18 h. Supernatants were collected and subjected to ELISA for the detection of soluble IFNγ secreted by activated T cells. Data summarise mean amounts of IFNγ of three mice analysed in technical triplicates. Statistical differences as indicated were determined using Mann-Whitney test.

To explore more precisely the role of AMPK in thymocyte positive selection we backcrossed AMPK^fl/fl^ CD4Cre^pos^ mice to mice expressing the defined OT1 αβ TCR transgene that select for class I restricted CD8 T cells. The data show that AMPK loss had no impact on the selection of thymocytes expressing the OT1 αβ TCR complex **(** **Figure 6A** **)**. It has been described that peripheral T cells from the whole body AMPKα1 null mice make higher levels of interferon γ (IFNγ) compared to wild type T cells [8]. The data in Figure 6B compare IFNγ production by naïve wild type and AMPKα1 null OT1 TCR transgenic T cells. These data show that deletion of AMPKα1 does cause enhanced production of IFNγ by CD8 T cells.

## Discussion

Previous studies have shown that LKB1 controls the survival of T cell progenitors at the DN stage of development. In these studies LKB1 was deleted at the DN2/3 stage of thymocyte development using the LckCre recombinase model. In LckCre LKB1^fl/fl^ mice a few DP thymocytes survive the early deletion of LKB1 and become DP thymocytes [6]. These DP thymocyte cells had a reduced survival capacity but it was unclear whether these results really inform as to whether LKB1 controls DP thymocyte survival. We have addressed this issue by using a CD4 promoter Cre recombinase to delete LKB1 directly in DP thymocytes. Our studies of LKB1 null DP thymocytes from the LKB1^fl/fl^ CD4Cre mice found no evidence that LKB1 was directly required for DP survival. LKB1 was however required for DPs to differentiate to become iNKT cells and for the production of conventional αβ T cells.

The role for LKB1 in the development of conventional αβ TCR T cells has been previously reported [6]–[9]. However, the present study now shows that LKB1 is also essential for the development of innate lymphoid populations such as iNKT cells and gut intraepithelial T cells. Moreover, the present study has more precisely delineated that LKB1 is not involved in the initial phase of positive selection of mature T cells. LKB1 null DPs can thus respond to TCR signals to up-regulate expression of CD69. However, they fail to complete positive selection to produce SP T cells in the thymus. Why would DPs fail to make iNKT cells? One explanation is that iNKT cell progenitors undergo a very robust proliferative expansion at the DP stage of thymocyte development. The basis for the failed iNKT cell development of LKB1 null DPs must thus reflect that LKB1 has an essential role for the proliferative burst of iNKT cells that accompanies the positive selection of these cells. This is reminiscent of the LKB1 requirement for the proliferative expansion of DN T cell progenitors and mature conventional T cells [6]. In this respect, previous studies [17] have shown that T cells undergoing positive selection undergo proliferative expansion once they up-regulate expression of CD69. These results are thus consistent with a model that LKB1 is necessary for T cells whenever there is a metabolic demand on the cells imposed by a phase of rapid proliferation. It is also noteworthy that a previous study has indicated that LKB1 might control the recruitment of phospholipase C-gamma 1 (PLCγ1) to the T cell membrane and hence directly control T cell antigen receptor signal transduction [9]. The failure of LKB1 null DP thymocytes to differentiate to iNKT cells could thus also reflect a failure of antigen receptor signalling in DP thymocytes. However, the ability of LKB1 null DPs to initiate positive selection and up-regulate CD69 expression is not consistent with a global defect in TCR-mediated signal transduction. Similarly, LKB1 null DPs express normal levels of CD5 (data not shown) and it is well established that CD5 expression in thymocytes is controlled by the strength of TCR signalling.

It has been proposed that LKB1 controls thymus development via its substrate AMPK [7], [8]. This model was first proposed because a constitutively active AMPK construct could ‘rescue’ the survival defects of LKB1 null thymocytes [7]. However, gain-of-function strategies do not always inform about the physiological role of a kinase and there has always been further more the contradictory data that whole body AMPKα1 null mice on mixed genetic background show normal T cell development. The caveat is that AMPKα1 null mice on a mixed genetic background are born at very low frequency [11]. Moreover, whole body deletion of AMPK results in embryonic lethality on a C57Bl/6 background (emmanet.org EM:0417). The question thus arises as to whether the failure to see a role of AMPKα1 in T cell development reflects that the few mice that survive AMPKα1 deletion have compensated for the loss of AMPKα1. Accordingly, to directly compare the impact of AMPKα1 deletion on thymocyte development the present study has used a CD4Cre transgene to delete AMPKαl floxed alleles at the DP stage of thymocyte development. Studies of CD4Cre AMPKα1^fl/fl^ mice show that AMPKα1 is not needed for the development of conventional αβ T cells or for the generation of innate-like lymphocytes, namely NKT cells and intraepithelial gut T lymphocytes. LKB1 thus controls the development of T cells in the thymus by AMPK independent mechanisms.

In this respect it is important to note that LKB1 phosphorylates and activates multiple members of the AMP-activated protein kinase (AMPK) subfamily such as the salt inducible kinase, the Par/MARK kinases and NUAK1 [18]. In this context, both the MARK kinases and NUAK1 have been proposed to regulate cell adhesion, polarity and migration in non-lymphoid cells. For example, the LKB1 effector kinase NUAK1 has recently been shown to regulate the activity of the myosin phosphatase MYPT1-PP1β complex leading to increased activity of myosin in fibroblasts [19]. Therefore, LKB1 potentially increases cell detachment by regulating the activity of NUAK1. We did therefore consider that LKB1 null DP thymocytes might show defects in integrin-dependent cell adhesion and migration. However, the present data show that the ability of LKB1 null cells to migrate in response to the chemo-attractant CXCL12 was unimpaired. We thus have no evidence that LKB1 regulates cell adhesion, polarity or migration of T cells. Nevertheless, the role of kinases such as NUAK1 in T cells will be interesting to explore because it has been reported that tumour cells exposed to energy stress undergo a NUAK/ARK5-dependent cell cycle arrest resulting in the protection from apoptosis: in the absence of NUAK1/ARK5, energy stress results in cell death potentially due to the lack of the inhibition of energy consuming processes [20]. The failure of LKB1 null T cells to complete positive selection could thus be caused by an important role for LKB1 and its substrates such as NUAK1 in returning positively selected thymocytes to a quiescent state.

## Materials and Methods

### Ethics Statement

Mice as outlined next were bred and maintained under specific pathogen-free conditions in the Biological Resource Unit at the University of Dundee. The procedures used were approved by the University Ethical Review Committee, a committee of the University Court, at its meeting on 19^th^ December 2007 and then authorised by a project licence under the UK Home Office Animals (Scientific Procedures) Act 1986 issued by the Home Office on 14^th^ April 2008.

### Mice

LKB1^fl/fl^ mice were generated and bred as previously described by Sakamoto *et al* [21]. LKB1^fl/fl^ mice were back-crossed to transgenic mice expressing *Cre* recombinase under the control of the human *cd4* promoter. AMPK^fl/fl^ mice were obtained from Benoit Viollet (Institut Cochin, INSERM, Université Paris Descartes, Paris) and bred to OT1-TCR transgenic mice and/or CD4Cre^+^ mice. Genotypes of bred mice were determined and confirmed by polymerase chain reaction of genomic DNA extracted from ear snips of weaned mice and expression of TCR transgene was confirmed by flow cytometric analysis of blood biopsies from the mouse tail veins. For tissue isolations with the exception of ear and blood biopsies for genotyping purposes, mice were sacrificed by increasing concentrations of carbon dioxide in compliance with the project licence.

### Cell Culture

Single cell suspensions of freshly isolated thymi were maintained at 5−10×10^6^ cells mL^−1^ in DMEM containing 10% heat-inactivated foetal calf serum (FCS) (Life Technologies, UK), 50 µM 2-mercaptoethanol (Sigma-Aldrich, Germany), 100 U mL^−1^ penicillin and 100 µg mL^−1^ streptomycin (Life Technologies, UK). Lymph nodes and spleens were gently disaggregated. Disaggregated spleens were also treated for lysis of red blood cells. Lymph nodes and spleens were re-suspended at 5−10×10^6^ cells mL^−1^ in RPMI-1640 containing L-glutamine and supplemented with 10% heat-inactivated FCS, 50 µM 2-mercaptoethanol, 100 U mL^−1^ penicillin and 100 µg mL^−1^ streptomycin. Primary CD8^pos^ T cells (4×10^6^ cells mL^−1^) from OT1-TCR transgenic mice were activated with 0.5 µM soluble ovalbumin-derived SIINFEKL peptide for 18 h in 96-well plates and supernatants were collected followed by cytokine secretion assays. IFNγ secretion was determined by enzyme-linked immunosorbent assay (ELISA) using a commercially available kit from eBiosciences.

### MACS Purification of double positive (DP) Thymocytes

Thymocytes (1×10^8^ cells) were labelled with biotinylated anti-CD4 (BD Pharmingen) and CD4^pos^ thymocytes were isolated using streptavidin-coated magnetic beads by autoMACS (Miltenyi Biotec, Germany). The positive fraction collected was then lysed for immunoblotting.

### Immunoblotting

Thymocytes (3×10^7^ cells) were lysed in 1 mL of F buffer [10 mM Tris-HCl pH 7.05, 50 mM NaCl, 30 mM Na-pyrophosphate, 50 mM NaF, 5 µM ZnCl_2_, 10% Glycerol, 1% NP-40, 1 mM DTT] supplemented with 50 nM calyculin A for 15 min on ice and centrifuged for 20 min at 1.32×10^4^ rpm. Lysates were mixed and boiled with NuPAGE LDS sample buffer (Life Technologies) supplemented with 100 mM DTT. Samples were separated on NuPAGE Bis-Tris 4–12% gradient gels (Life Technologies) at 200 V for up to 60 min under reducing conditions. Separated proteins were transferred onto Hybond™-C Super nitrocellulose membrane (Amersham Biosciences, UK) at 30 V for 150 min in Novex XCell II Modules (Invitrogen, UK) at 4°C. Membranes were blocked with 5% dry milk/PBS supplemented with 0.5% Tween-20 (Sigma) and probed for indicated pan-proteins. Anti-AMPKα1 was a kind gift of Grahame Hardie, University of Dundee. Anti-Smc1 was obtained from Bethyl Laboratories Inc. All other antibodies for immunoblotting were obtained from Cell Signaling Technology.

### Isolation of intraepithelial gut lymphocytes

Freshly isolated small intestines were freed from mesenteric lymph nodes, Peyer's patches, debris, adipose and connective tissues. Digested food was removed mechanically and the intestinal lumen was cleaned using PBS. Intestines were opened longitudinally and then cut into 1-cm-pieces, which were incubated in Ca^2+^/Mg^2+^-free PBS (Sigma) supplemented with 10% filtered heat-inactivated FCS, 1 mM Na pyruvate, 20 mM HEPES pH 8.0, 10 mM EDTA pH 8.0 and 10 µg mL^−1^ Polymyxin B for 30 min at 230 rpm and 37 °C. Tissue suspensions were filtered using a 70 µm-filter cell strainer (BD Falcon) and cells were collected by centrifugation. Cells were re-suspended in 37.5% isotonic percoll (Sigma) and collected by centrifugation (without break). Following careful recovery of the cell pellet, cells were washed, re-suspended in complete RPMI-1640 culture medium, filtered through a 40- µm-filter (BD Falcon) and stained for flow cytometric analysis.

### Flow cytometry

Accurate cell counts of lymphocyte cultures were taken by using AccuCheck counting beads (Life Technologies, UK). One to two million cells of freshly disaggregated secondary lymphoid organs or extracted from small intestines were incubated with F_C_ Block (BD Pharmingen) for 10 min at 4°C in RPMI-1640 or PBS supplemented with 1% FCS (FACS buffer). F_C_ Block was omitted for thymocyte suspensions. Cells were labelled with saturating concentrations of antibody in FACS buffer. Antibodies used were conjugated to fluorescein-isothiocyanate, phycoerythrin (PE), peridinin-chlorophyll protein (PerCP)-Cy.5.5, PE-Cy7, allophycocyanin (APC), APC-Cy7 or –eFluor®780, Horizon V450 or V500, Alexa Fluor®700 as obtained from BD Pharmingen or eBiosciences: anti-CD4 (L3T4), anti-CD8α (53-6.7), anti-CD8β (H35-17.2), anti-CD44 (IM7), anti-CD69 (H1.2F3), anti-Vα2 TCR (B20.1), anti-Vβ5.1/5.2 TCR (MR9-4), anti-TCRβ (H57-597), anti-TCRγδ (GL3) and anti-CD24 (M1/69). Staining for Vα14 TCR to detect NKT cells was performed as described previously [8], [9], [14]. Where mentioned, DAPI was used at a concentration of 1 µg mL^−1^ for live cell determination. Following incubation with antibodies, cells were washed and resuspended in FACS buffer. Samples were analysed using a FACSCalibur, LSR II Fortessa or Canto (Becton Dickinson). A minimum of 1×10^4^ ungated events were acquired and stored. Data files were processed using the latest version of FlowJo software V9.6 (Treestar) for Mac OS. Live cells were gated according to their forward and side scatters and exclusion of DAPI, where indicated.

### Transwell migration assay

Migration assays were performed using Transwell chemotaxis plates (CoStar). Membrane inserts of transwell plates were coated with 5 µg mL^−1^ fibronectin at 4 °C over night. Membranes were then blocked with 2% heat-inactivated FCS/PBS for one hour at 37 °C. Freshly isolated thymocytes (1×10^6^ cells in 100 µL of complete DMEM medium) were placed in the upper chamber of the transwell plate in triplicate. Culture medium without or with CXCL12 (500 ng mL^−1^ in 600 µL) was placed in the lower chamber. After 3 h of incubation at 37 °C in 5% CO_2_, the percentage of cells against the input control was determined using flow cytometry.

### Statistical Analysis

Quantified data were evaluated using non-parametric Mann-Whitney test, where experimental numbers were not sufficient to prove normal distribution. Bar graphs are shown as mean ± standard deviation unless otherwise stated. GraphPad Prism 4.0c or later for Mac OS X was used for statistical evaluation and generation of bar graphs and dot plots of quantified data.
